# Supplementary material for: Emotion regulation or dual task? Dissociation of neural and behavioral measures
Source: bioRxiv. 2026 Apr 21:2026.04.17.719189. Preprint. [Version 1] doi: 10.64898/2026.04.17.719189 (PMC13131790; doi:10.64898/2026.04.17.719189)
Supplement: 1 [file NIHPP2026.04.17.719189V1-supplement-1.pdf]

bioRxiv preprint doi: https://doi.org/10.64898/2026.04.17.719189; this version posted April 21, 2026. The copyright holder for this preprint (which was not certified by peer review) is the author/funder, who has granted bioRxiv a license to display the preprint in perpetuity. It is made available under aCC-BY 4.0 International license.

SUPPLEMENTARY INFORMATION

Supplementary Methods

Regulation instruction wording

Participants were trained on three instruction conditions prior to the task. The following wording was used:

View: "Simply look at the picture."

Enhance: "We would like you to increase the intensity of the emotion you feel in response to the picture. Try to feel the emotion more strongly. Do not generate thoughts and images that are completely unrelated to the picture in order to produce a different emotion. However, you may focus on a specific feature of the picture or think of an outcome of the situation depicted in the picture that will help to enhance your emotion. Prepare yourself to feel the emotion more strongly."

Suppress: "We would like you to decrease the intensity of the emotion you feel in response to the picture. Try to feel the emotion less strongly. Suppression of an emotion is not the same as replacing that emotion with a different one. Do not generate thoughts and images that are completely unrelated to the picture in order to produce a different emotion. However, feel free to focus on a positive aspect of the picture or on a possible positive outcome of the situation in the picture. Prepare yourself to feel the emotion less strongly."

Participants practiced each instruction with feedback from the experimenter until they could provide at least two adequate examples of each strategy

Rationale for combining samples

Given the small-to-medium effect sizes typically reported for cognitive reappraisal modulations of the LPP (Zou et al., 2026), the two samples were combined to maximize statistical power. Monte Carlo simulations on LPP data collected with the same recording system used here have shown that, for within-subject effect sizes below 1 μV, achieving 80% statistical power requires at least 50 participants and that adding subjects increases power more effectively than adding trials (Gibney et al., 2020). Because the instruction effects of interest fall well below 1 μV, neither Sample 1 (N = 73) nor Sample 2 (N = 34) alone provides adequate power, particularly for the critical Enhance versus Suppress contrast. Combining was contingent on two prerequisites: (a) a sensitivity power analysis confirming inadequate individual power, and (b) a mixed-model ANOVA confirming equivalent LPP responses across samples. Both conditions were met (see below).

bioRxiv preprint doi: https://doi.org/10.64898/2026.04.17.719189; this version posted April 21, 2026. The copyright holder for this preprint (which was not certified by peer review) is the author/funder, who has granted bioRxiv a license to display the preprint in perpetuity. It is made available under aCC-BY 4.0 International license.

EEG preprocessing details

Offline, continuous EEG data were visually inspected, and broken channels were interpolated using spherical splines. After re-referencing to the average of all electrodes, eye blinks were corrected using a spatiotemporal filtering method as implemented in BESA 7.1.2.1 (BESA GmbH, Gräfelfing, Germany). Subsequent analyses were performed in BrainVision Analyzer 2.3.0 (Brain Products GmbH, Gilching, Germany). Data were bandpass-filtered to 0.1-40 Hz and segmented into 1100 ms epochs time-locked to picture onset. Epochs were baseline-corrected using the 100 ms pre-stimulus interval. Automated artifact detection was applied to each channel to identify epochs contaminated by artifacts. The following criteria defined artifacts: EEG amplitude above 100 or below -100 µV during the epoch; absolute voltage difference between any two data points within the segment larger than 100 µV; voltage difference between two contiguous data points above 25 mV and less than 0.5 µV variation for more than 100 ms. Channels contaminated by artifacts in more than 40% of the epochs were interpolated using spherical splines. Epochs with more than 10% of channels contaminated by artifacts were discarded. After these steps, ERPs were calculated for each channel and each condition.

Detailed statistical procedures

Emotional intensity ratings. Trial-level emotional intensity ratings (1–7 scale) were averaged within each Valence × Instruction cell for each participant. A 2 (Valence: Pleasant, Unpleasant) × 3 (Instruction: View, Enhance, Suppress) repeated-measures ANOVA tested whether regulation instructions modulated subjective experience. The emotion effect was tested separately with a one-way RM ANOVA on View-condition ratings (Neutral, Pleasant, Unpleasant). All ANOVAs used Greenhouse-Geisser correction where sphericity was violated. Follow-up pairwise comparisons used paired t-tests with Cohen's d as the within-subject effect size. Cigarette-related pictures were analyzed separately.

LPP: Emotion effect. To confirm LPP sensitivity to emotional content, we tested the effect of picture valence in the View condition only using a one-way repeated-measures ANOVA with Valence (Pleasant, Neutral, Unpleasant). The Greenhouse-Geisser correction was applied when sphericity was violated. Follow-up comparisons contrasted each emotional category against Neutral using two-tailed paired t-tests. Effect sizes are reported as Cohen's d with 95% confidence intervals.

LPP: Reappraisal model testing. A 2 × 3 repeated-measures ANOVA with Valence (Pleasant, Unpleasant) and Instruction (View, Enhance, Suppress) was conducted. The standard paradigm introduces a confound: View is a single-task condition, whereas Enhance and Suppress are dual-task conditions. To adjudicate between accounts, we adopted a Bayesian model-comparison framework testing two orthogonal contrasts:

bioRxiv preprint doi: https://doi.org/10.64898/2026.04.17.719189; this version posted April 21, 2026. The copyright holder for this preprint (which was not certified by peer review) is the author/funder, who has granted bioRxiv a license to display the preprint in perpetuity. It is made available under aCC-BY 4.0 International license.

(1) Reappraisal model (Enhance − Suppress, [0, +1, −1]). If participants can volitionally regulate the LPP, Enhance should exceed Suppress. (2) Cognitive load model (2×View − Enhance − Suppress, [+2, −1, −1]). If both regulation instructions divert attentional resources, both should reduce the LPP equally relative to View. These contrasts are mathematically orthogonal and provide independent tests. For each, we computed a one-sample t-test against zero on subject-level contrast scores (collapsed across valences) and derived BF_{01} using the JZS prior (Cauchy scale $r = \sqrt{2}/2 \approx 0.707$; Rouder et al., 2009) as implemented in the pingouin library.

LPP: Robustness analyses. Two complementary checks were performed. First, a sequential Bayes Factor analysis evaluated evidence accumulation across participants. Subjects were randomly ordered (500 permutations), and BF_{01} for both contrasts was computed at incremental sample sizes (N = 20, 25, ..., 107). Median BF_{01} values and 90% credible intervals are reported. Second, a nonparametric bootstrap analysis (10,000 resamples) estimated the sampling distribution of Cohen's d for each contrast.

LPP: Arousal × Instruction analysis. We examined whether instruction effects varied with stimulus arousal. The original stimulus set included high-arousal and low-arousal subcategories within each valence. We compared View to a collapsed Regulate condition (mean of Enhance and Suppress) separately for high- and low-arousal pictures. Bayesian t-tests were computed for both the Reappraisal and View vs. Regulate contrasts at each arousal level.

Multivariate pattern analysis. To test whether instruction-related signals are present anywhere on the scalp, we applied multivariate pattern analysis (MVPA) using a two-step positive-control approach. The same pipeline was used for: (1) emotion decoding (Emotional vs. Neutral, View condition; positive control), and (2) instruction decoding (Enhance vs. Suppress, emotional pictures; critical test). For emotion classification, the Emotional class was the average of Pleasant-High and Unpleasant-High conditions under View; Neutral was the Neutral-View condition. For instruction classification, Enhance was the average of Pleasant-Enhance and Unpleasant-Enhance; Suppress was the average of Pleasant-Suppress and Unpleasant-Suppress. Each subject contributed one observation per class.

Feature vectors comprised mean voltage at all 129 channels within each of 20 consecutive 50-ms time bins spanning 0–1000 ms, yielding 2,580 features per observation. The pipeline consisted of z-score standardization, PCA retaining 95% of variance, and L2-regularized logistic regression (C = 1.0), with all transformations fit on training data only within each cross-validation fold. Leave-one-subject-out cross-validation (107 folds) was used. Statistical significance was assessed by permutation testing (1,000 iterations). Time-resolved analysis trained separate classifiers at each 50 ms bin using standardization and logistic regression without PCA, with permutation testing (100 iterations per bin).

bioRxiv preprint doi: https://doi.org/10.64898/2026.04.17.719189; this version posted April 21, 2026. The copyright holder for this preprint (which was not certified by peer review) is the author/funder, who has granted bioRxiv a license to display the preprint in perpetuity. It is made available under aCC-BY 4.0 International license.

Supplementary Results

Sensitivity Power Analysis

To evaluate whether pooling the two samples was warranted, we conducted a sensitivity power analysis for paired *t*-tests across a range of Cohen's *d* values (0.05 to 0.80) and sample sizes (10 to 300), with alpha = .05 (two-tailed). Power was computed analytically using the non-central *t* distribution. The observed effect sizes from the pooled sample were then projected onto these curves to determine the power available at each sample size (Supplementary Figure 1).

For the basic emotion effect (emotional vs. neutral pictures under passive viewing), the observed effect sizes were medium in magnitude (*d* = 0.41 for pleasant, *d* = 0.49 for unpleasant). Sample 1 (*N* = 73) was adequately powered for both contrasts (power = .93 and .99, respectively), whereas Sample 2 (*N* = 34) reached 80% power only for the unpleasant contrast. The critical effects for the present study, however, are the instruction contrasts. The observed cognitive load effect (View vs. Regulation) yielded *d* = 0.257, and the individual pairwise contrasts (View vs. Enhance, View vs. Suppress) ranged from *d* = 0.236 to 0.261. At these effect sizes, Sample 2 afforded only 27-32% power and Sample 1 only 51-59% power, both well below the conventional 80% threshold. Achieving 80% power for these contrasts requires between 118 and 143 participants, confirming the need for pooling. Notably, the Enhance versus Suppress contrast, the key test of the reappraisal account, yielded a near-zero observed effect (*d* = 0.030), which would require 80% power with over 8,700 participants, reinforcing the conclusion that the absence of a reappraisal effect is not attributable to insufficient sample size. These results converge with Monte Carlo simulations conducted on LPP data collected with the same recording system, which demonstrated that within-subject effects below 1 μV require at least 50 participants to reach adequate power.

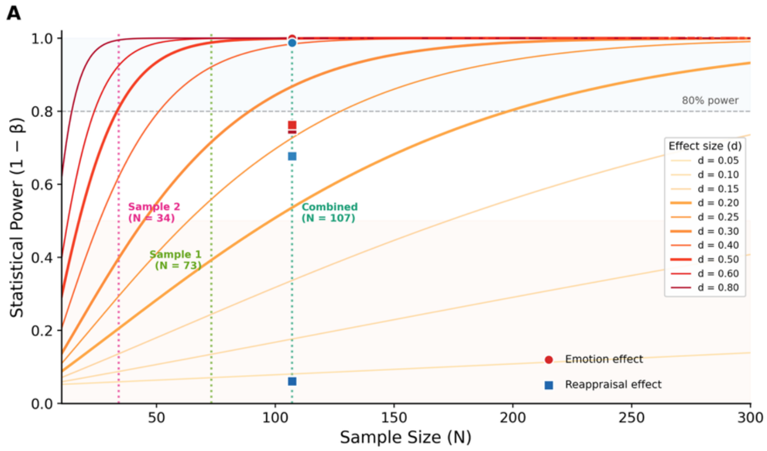

B

Power at each sample size (α = .05, two-tailed)

Effect	d	Power N=34	Power N=73	Power N=107	N for 80%
Unpleasant > Neutral	0.493	0.80	0.99	1.00	35
Pleasant > Neutral	0.410	0.64	0.93	0.99	49
Cognitive load (2V−E−S)	0.257	0.31	0.58	0.75	121
View vs. Suppress	0.261	0.32	0.59	0.76	118
View vs. Enhance	0.236	0.27	0.51	0.68	143
Reappraisal (E−S)	0.030	0.05	0.06	0.06	8723

bioRxiv preprint doi: https://doi.org/10.64898/2026.04.17.719189; this version posted April 21, 2026. The copyright holder for this preprint (which was not certified by peer review) is the author/funder, who has granted bioRxiv a license to display the preprint in perpetuity. It is made available under aCC-BY 4.0 International license.

Supplementary Figure 1. Sensitivity power analysis justifying sample pooling. (A) Statistical power ($1 - \beta$) as a function of sample size for paired *t*-tests at effect sizes ranging from $d = 0.05$ to $d = 0.80$ ($\alpha = .05$, two-tailed). Vertical dotted lines indicate Sample 2 ($N = 34$), Sample 1 ($N = 73$), and the combined sample ($N = 107$). Colored markers show observed effect sizes from the pooled analysis projected onto the combined sample size: circles denote emotion effects (emotional vs. neutral, View condition) and squares denote instruction effects. The dashed horizontal line marks 80% power. (B) Power estimates for each observed effect at each sample size, with the sample size required to achieve 80% power. Green cells indicate adequate power ($\geq .80$), yellow cells marginal power (.50 to .79), and red cells insufficient power ($< .50$).

Cohort Equivalence

To confirm that the two samples produced comparable LPP responses, we conducted a mixed-model ANOVA on LPP amplitudes in the View condition, with Valence (Pleasant, Unpleasant, Neutral, Cigarette) as a within-subjects factor and Cohort (Sample 1, Sample 2) as a between-subjects factor. The two samples did not differ in overall LPP amplitude, $F(1, 105) = 2.61$, $p = .109$, $\eta^2p = .024$, and the Valence × Cohort interaction was not significant, $F(3, 315) = 0.52$, $p = .667$, $\eta^2p = .005$, indicating that the pattern of valence-dependent LPP modulation was consistent across samples. The main effect of Valence was significant, $F(3, 315) = 12.58$, $p < .001$, $\eta^2p = .107$, confirming that the basic emotion effect replicated in both samples. Having satisfied both prerequisites for pooling (insufficient power in individual samples and equivalent LPP responses across cohorts), all subsequent analyses were conducted on the combined sample ($N = 107$).

Regulation Effect in Self-Report Ratings

To test whether behavioral regulation varied with stimulus intensity, we conducted separate 2 (Arousal: High, Low) × 3 (Instruction: View, Enhance, Suppress) repeated-measures ANOVAs for pleasant and unpleasant pictures (Supplementary Fig. 2). For pleasant pictures, both main effects were significant, Arousal, $F(1, 105) = 96.69$, $p < .001$, $\eta^2G = .081$, and Instruction, $F(2, 210) = 56.84$, $p < .001$, $\eta^2G = .062$, $\varepsilon = .70$, with no Arousal × Instruction interaction, $F(2, 210) = 2.18$, $p = .115$, indicating that regulation effects were comparable across arousal levels. For unpleasant pictures, both main effects were again significant, Arousal, $F(1, 105) = 103.38$, $p < .001$, $\eta^2G = .112$, and Instruction, $F(2, 210) = 49.12$, $p < .001$, $\eta^2G = .046$, $\varepsilon = .77$, and the interaction reached significance, $F(2, 210) = 5.92$, $p = .003$, reflecting a smaller enhancement effect for high-arousal ($d = 0.32$) than low-arousal ($d = 0.67$) unpleasant pictures. Critically, all 12 pairwise contrasts were significant (all *p*s < .01, $|d| = 0.32$–0.82; **Fig. 2F**), confirming that behavioral regulation was effective at both arousal levels for both valence categories.

bioRxiv preprint doi: https://doi.org/10.64898/2026.04.17.719189; this version posted April 21, 2026. The copyright holder for this preprint (which was not certified by peer review) is the author/funder, who has granted bioRxiv a license to display the preprint in perpetuity. It is made available under aCC-BY 4.0 International license.

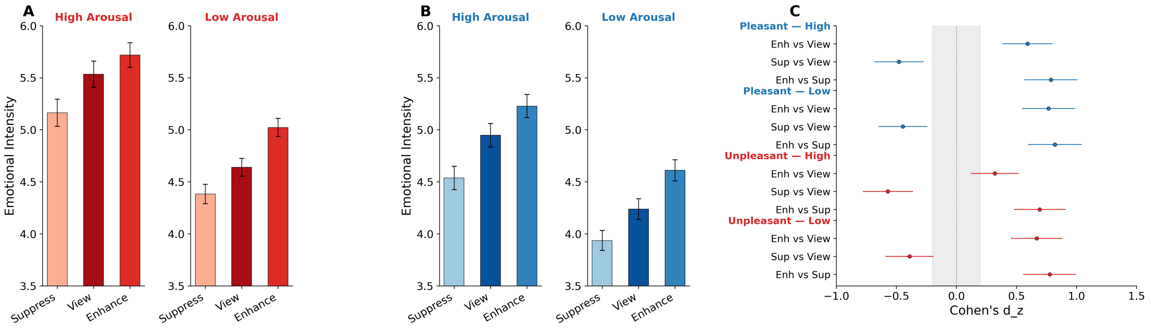

Supplementary Figure 2. (**A**) Mean ratings for pleasant pictures by arousal level (High, Low) and instruction condition. (**B**) Mean ratings for unpleasant pictures by arousal level and instruction condition. Regulation effects were significant at both arousal levels for both valence categories (all *p*s < .01). (**C**) Forest plot of arousal-specific within-subject effect sizes. **p < .01, ***p < .001.

First View: Emotion Effect Prior to Instruction

To verify that the LPP was sensitive to emotional content before any regulatory instructions were introduced, we examined the first picture presentation, during which all pictures were passively viewed regardless of the upcoming instruction condition. Grand-average ERP waveforms showed clear differentiation among all four valence categories beginning approximately 200 ms post-stimulus, with pleasant and unpleasant pictures producing the largest sustained positivity across the LPP window (Supplementary Fig. 3A). Cigarette pictures tracked between emotional and neutral conditions throughout the epoch. Topographic maps of the Emotional − Neutral difference confirmed a centro-parietal distribution emerging by 200–400 ms and strengthening through the LPP window (Supplementary Fig. 2B, top row), and individual valence topomaps at 400–800 ms showed the expected pattern of broadly distributed positivity for all stimulus categories relative to neutral (Supplementary Fig. 3B, bottom row).

A one-way repeated-measures ANOVA on mean LPP amplitude (400–800 ms) with Valence (Pleasant, Neutral, Unpleasant, Cigarette) confirmed a significant main effect, $F(3, 318) = 71.39$, $p < .001$, $\eta^2G = .123$, $\varepsilon = .86$ (Supplementary Fig. 3C). All three stimulus categories elicited significantly larger LPPs than Neutral: Pleasant, $t(106) = 12.87$, $p < .001$, $d = 1.24$ [1.00, 1.50]; Unpleasant, $t(105) = 9.89$, $p < .001$, $d = 0.96$ [0.73, 1.19]; and Cigarette, $t(106) = 5.76$, $p < .001$, $d = 0.56$ [0.35, 0.76]. Pleasant pictures also elicited larger LPPs than Unpleasant pictures, $t(105) = 6.39$, $p < .001$, $d = 0.62$ [0.41, 0.83].

bioRxiv preprint doi: https://doi.org/10.64898/2026.04.17.719189; this version posted April 21, 2026. The copyright holder for this preprint (which was not certified by peer review) is the author/funder, who has granted bioRxiv a license to display the preprint in perpetuity. It is made available under aCC-BY 4.0 International license.

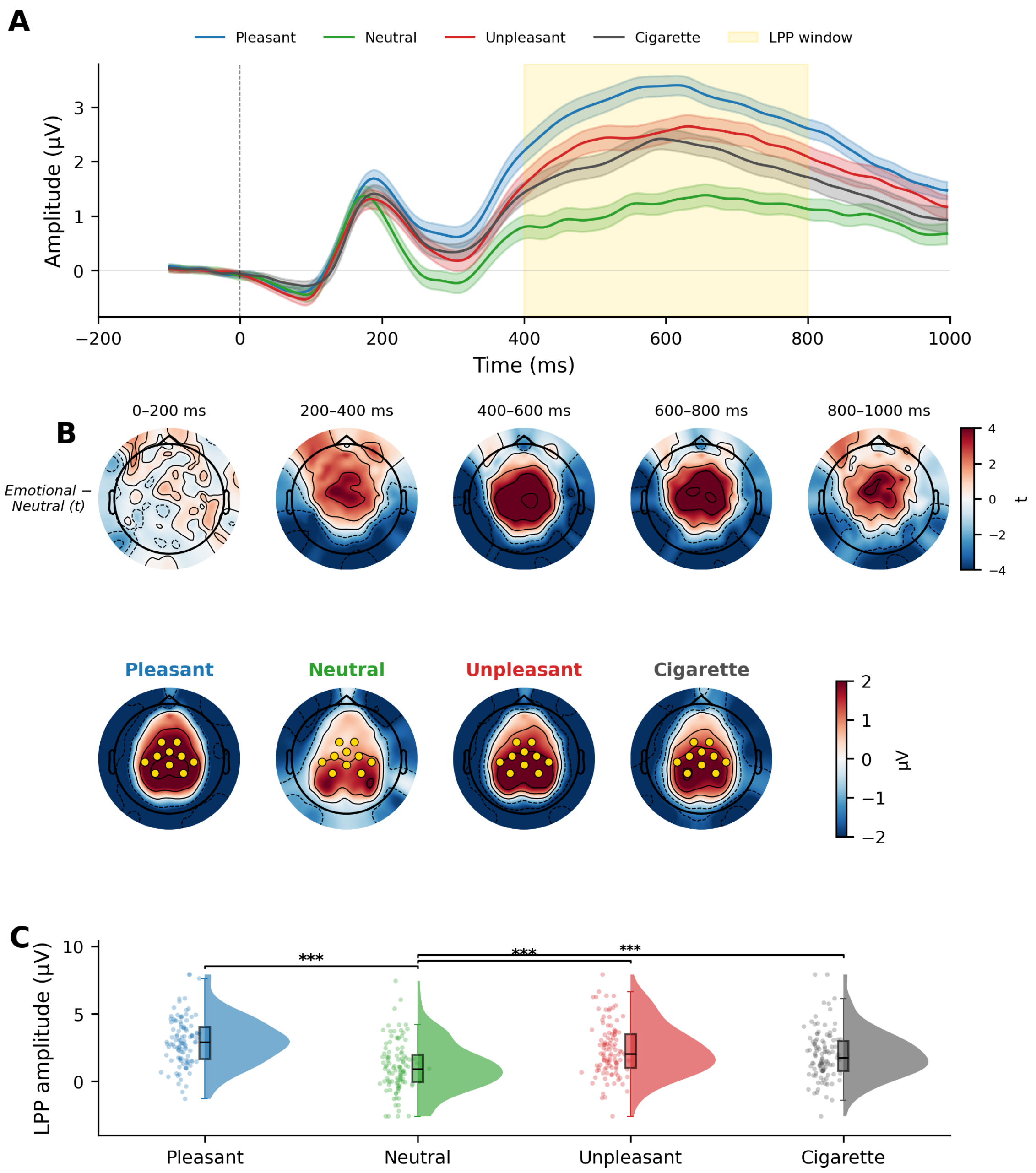

Supplementary Figure 3. Emotional pictures elicit larger late positive potentials during the first (pre-instruction) picture presentation. (**A**) Grand-average ERP waveforms at centro-parietal ROI channels for pleasant (blue), neutral (green), unpleasant (red), and cigarette (gray) pictures during the first passive viewing. Shaded ribbons represent ±1 SEM. Yellow shading indicates the LPP measurement window (400–800 ms). (**B**) Top row: topographic maps of the Emotional − Neutral difference (*t*-statistic) across five 200-ms time bins, showing the emergence of the centro-parietal emotion effect. Bottom row: topographic maps of mean scalp voltage (µV) during the LPP window (400–800 ms) for each valence category. Gold dots indicate a priori ROI channels. (**C**) Raincloud plots of individual LPP amplitudes by valence category. All three stimulus categories elicited significantly larger LPPs than Neutral (all *ps* $< .001$). ***p $< .001$.

bioRxiv preprint doi: https://doi.org/10.64898/2026.04.17.719189; this version posted April 21, 2026. The copyright holder for this preprint (which was not certified by peer review) is the author/funder, who has granted bioRxiv a license to display the preprint in perpetuity. It is made available under aCC-BY 4.0 International license.

Arousal modulates the cognitive load effect.

Given the absence of any direction-specific instruction effect in the primary analysis (Enhance vs. Suppress BF01 = 9.30), the following analyses collapse Enhance and Suppress into a single Regulate condition to examine whether the overall attentional load effect varies with stimulus arousal. As a confirmatory check, Enhance and Suppress were also examined separately; the two conditions did not differ at either arousal level for either valence category (all BF01s > 3.5), justifying the collapsed approach. To test whether the cognitive load effect varied with stimulus intensity, we exploited the within-design arousal manipulation by comparing high-arousal and low-arousal stimuli separately. In the View condition (Supplementary Fig. 4A), pictures pre-selected to be included in the high-arousal category elicited substantially larger LPPs than low-arousal pictures, which in turn elicited larger LPPs than Neutral (all ps < .05). We then compared View to a collapsed Regulate condition (average of Enhance and Suppress) at each arousal level (Supplementary Fig. 4B). For high-arousal pictures, View produced significantly larger LPPs than Regulate for both pleasant and unpleasant stimuli (both ps < .05). For low-arousal pictures, the difference was small and nonsignificant. Critically, this outcome is inconsistent with the predictions of both the strong-situation and strategy-selection frameworks. Both frameworks predict that instruction-specific LPP modulation, that is, the Enhance > View > Suppress ordering, should be most evident at low arousal, when participants can fully implement the reappraisal strategy and volitional control is least constrained by stimulus intensity. Yet no instruction effect in any direction emerged at low arousal. The cognitive load pattern appeared exclusively at high arousal, where the emotional LPP is large enough to make attentional competition detectable.

Bayesian analyses revealed a complementary dissociation (Supplementary Fig. 4C). The Reappraisal contrast (Enhance − Suppress) yielded inconclusive-to-null evidence at both arousal levels, confirming that regulation direction did not modulate the LPP regardless of stimulus intensity. The View vs. Regulate contrast showed strong evidence for the alternative at high arousal (View > Regulate) but shifted toward the null at low arousal.

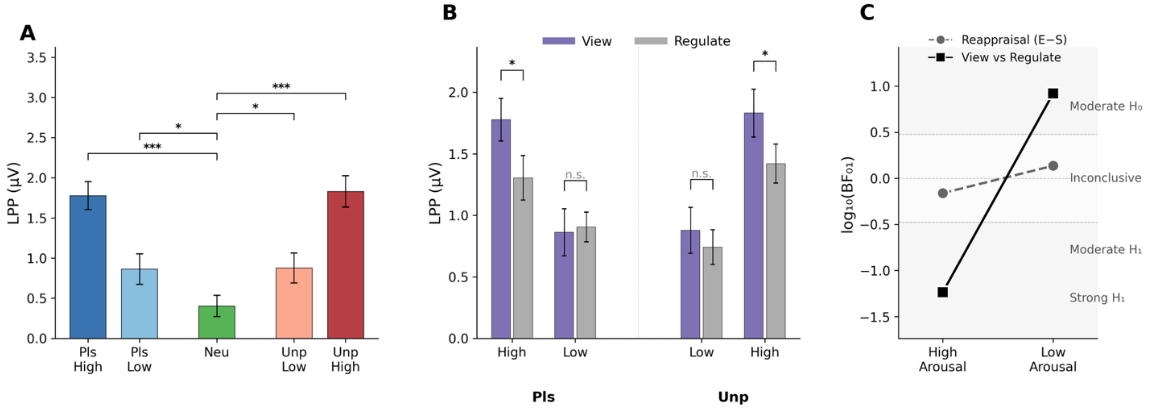

bioRxiv preprint doi: https://doi.org/10.64898/2026.04.17.719189; this version posted April 21, 2026. The copyright holder for this preprint (which was not certified by peer review) is the author/funder, who has granted bioRxiv a license to display the preprint in perpetuity. It is made available under aCC-BY 4.0 International license.

Supplementary Figure 4. The cognitive load effect on the LPP is arousal-dependent. (A) Mean LPP amplitude (±SEM) during passive viewing for high- and low-arousal pictures. (B) Instruction effects (View vs. Regulate) for each arousal × valence cell. (C) Bayesian evidence slope graph.

Cigarette Pictures

Behavioral ratings

In the View condition, cigarette pictures were rated as more emotionally intense than neutral pictures, $t(106) = 4.98$, $p < .001$, $d = 0.48$, but lower than both pleasant, $t(106) = -2.78$, $p = .006$, $d = -0.27$, and unpleasant pictures, $t(106) = -6.06$, $p < .001$, $d = -0.59$ (Supplementary Fig. 5A). Regulation instructions modulated cigarette ratings in the same direction as emotional pictures. A one-way repeated-measures ANOVA showed a significant effect of Instruction, $F(2, 212) = 48.72$, $p < .001$, $\eta G^2 = .043$, $\varepsilon = .75$. Enhance instructions increased ratings relative to View, $t(106) = 9.40$, $p < .001$, $d = 0.91$, and Suppress instructions decreased them, $t(106) = -3.10$, $p = .003$, $d = -0.30$ (Supplementary Fig. 5B). The Enhance–Suppress difference was large, $t(106) = 7.92$, $p < .001$, $d = 0.77$. These behavioral effects parallel the pattern observed for pleasant and unpleasant pictures in the main analysis, confirming that participants applied the regulation instructions consistently across all stimulus categories.

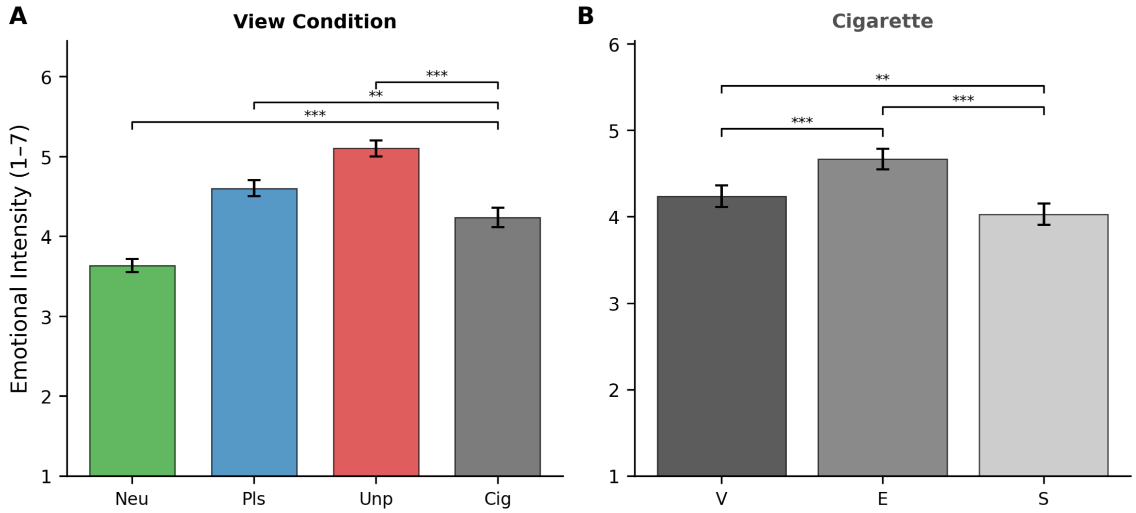

Supplementary Figure 5. Behavioral ratings for cigarette-related pictures. (A) Mean emotional intensity ratings in the View condition across all four stimulus categories. Cigarette pictures were rated as more intense than neutral ($p < .001$, $d = 0.48$) but less intense than both pleasant ($p = .006$, $d = -0.27$) and unpleasant pictures ($p < .001$, $d = -0.59$). (B) Mean ratings for cigarette pictures under View, Enhance, and Suppress instructions. The instruction effect paralleled the pattern observed for emotional pictures, with Enhance increasing and Suppress decreasing ratings relative to View. Error bars represent ±1 SEM. **$p < .01$, ***$p < .001$.

bioRxiv preprint doi: https://doi.org/10.64898/2026.04.17.719189; this version posted April 21, 2026. The copyright holder for this preprint (which was not certified by peer review) is the author/funder, who has granted bioRxiv a license to display the preprint in perpetuity. It is made available under aCC-BY 4.0 International license.

Late Positive Potential in the second presentation

In the pooled sample, cigarette pictures viewed passively ($M = 0.46$, $SD = 1.65$ μV) did not differ from neutral pictures ($M = 0.37$, $SD = 1.43$ μV), $t(106) = 0.54$, $p = .588$, $d = 0.05$, 95% CI [−0.14, 0.24] (Supplementary Fig. 6A). This null effect was consistent across samples: Sample 1, $t(72) = 0.73$, $p = .465$, $d = 0.09$; Sample 2, $t(33) = -0.12$, $p = .907$, $d = -0.02$. Grand-average waveforms confirmed that the cigarette time course tracked the neutral condition throughout the LPP window (400–800 ms), in contrast to the clear divergence of pleasant and unpleasant waveforms from neutral beginning around 300 ms (Supplementary Figure 6A).

Reappraisal instructions did not modulate LPP amplitude to cigarette pictures (Supplementary Fig. 6B). In the pooled sample, descriptive statistics were: View ($M = 0.46$, $SD = 1.65$ μV), Enhance ($M = 0.60$, $SD = 1.33$ μV), and Suppress ($M = 0.49$, $SD = 1.58$ μV). Pairwise comparisons were uniformly nonsignificant: View vs. Enhance, $t(106) = -0.92$, $p = .358$, $d = -0.09$ [−0.28, 0.10]; View vs. Suppress, $t(106) = -0.21$, $p = .835$, $d = -0.02$ [−0.21, 0.17] (Supplementary Figure 3B). This pattern was consistent in both cohorts: in the Discovery sample ($N = 73$), View vs. Enhance: $t(72) = -1.23$, $p = .222$, $d = -0.15$; View vs. Suppress: $t(72) = -0.43$, $p = .667$, $d = -0.05$; in the Replication sample ($N = 34$), View vs. Enhance: $t(33) = -0.01$, $p = .994$; View vs. Suppress: $t(33) = 0.34$, $p = .737$.

Bayesian model comparison confirmed the absence of any instruction effect for cigarette pictures (Supplementary Fig. 6C). The Reappraisal contrast (Enhance − Suppress) yielded $BF_{01} = 7.5$, indicating moderate evidence for the null. The Cognitive Load contrast (2 × View − Enhance − Suppress) yielded $BF_{01} = 7.5$, similarly favoring the null. The combined Null model received very strong support ($BF_{01} = 56.1$). This pattern was consistent across samples (Discovery: combined null $BF_{01} = 32.3$; Replication: $BF_{01} = 28.4$). The absence of both emotion and instruction effects for cigarette pictures is internally consistent. Because cigarette images did not engage the motivational systems indexed by the LPP, there was no emotional response to modulate with reappraisal instructions, nor differential attentional capture to reduce cognitive load. This null pattern serves as an informative negative control, demonstrating that the instruction effects observed for pleasant and unpleasant stimuli were not an artifact of task demand or nonspecific compliance.

Since visual inspection of the grand-average waveforms (Supplementary Figure 3A) suggested an early modulatory effect for cigarette pictures relative to neutral, we conducted a sub-window analysis, splitting the LPP epoch into early (400–600 ms) and late (600–800 ms) halves. The Cigarette–Neutral difference was nonsignificant in both sub-windows (400–600 ms: $t(106) = 1.77$, $p = .080$, $d = 0.17$, $BF_{01} = 5.6$; 600–800 ms: $t(106) = 0.13$, $p = .898$, $d = 0.01$, $BF_{01} = 25.8$), whereas Pleasant and Unpleasant pictures remained significantly above Neutral in both sub-windows (all $ps < .001$, $ds = 0.48–0.67$). The

bioRxiv preprint doi: https://doi.org/10.64898/2026.04.17.719189; this version posted April 21, 2026. The copyright holder for this preprint (which was not certified by peer review) is the author/funder, who has granted bioRxiv a license to display the preprint in perpetuity. It is made available under aCC-BY 4.0 International license.

apparent early divergence thus reflects a transient, unreliable deflection rather than sustained motivational engagement, as indexed by the LPP.

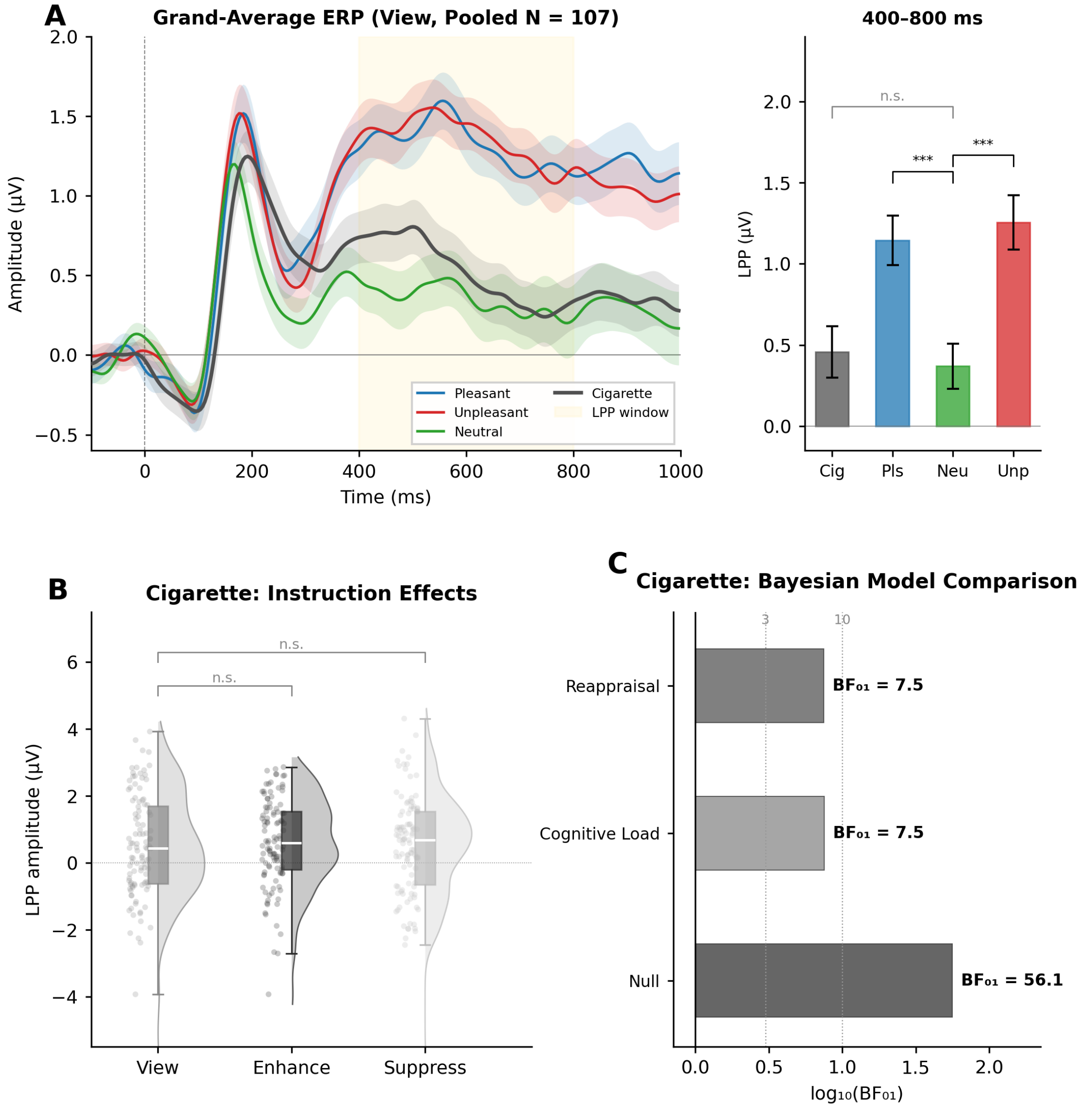

Supplementary Figure 6. Cigarette-related stimuli: LPP results. (A) Grand-average ERP waveforms at centro-parietal ROI channels in the View condition for all four stimulus categories (left) and mean LPP amplitude (400–800 ms) with ±1 SEM error bars (right). Cigarette pictures did not differ from the Neutral condition. ***p < .001, n.s. = not significant. **(B)** Raincloud plots of LPP amplitude for cigarette pictures under View, Enhance, and Suppress instructions. No pairwise comparisons reached significance. **(C)** Bayesian model comparison for cigarette pictures. All three models favor the null hypothesis, with the combined Null model receiving very strong support (BF_{01} = 56.1). Dotted lines indicate BF = 3 and BF = 10 evidence thresholds.
